# Medicinal plants, traditional medicine, markets and management in far-west Nepal

**DOI:** 10.1186/1746-4269-9-24

**Published:** 2013-04-12

**Authors:** Ripu M Kunwar, Laxmi Mahat, Ram P Acharya, Rainer W Bussmann

**Affiliations:** 1 Center for Biological Conservation, Kathmandu, Nepal; 2 Central Department of Sociology/Anthropology, Tribhuvan University, Kathmandu, Nepal; 3 Practical Solution Consultancy, Kathmandu, Nepal; 4 William L. Brown Center, Missouri Botanical Garden, P.O. Box 299, St. Louis, MO, 63166-0299, USA

**Keywords:** Medicinal plants, Indigenous use, Sustainable management, *Baidhya*, Baitadi district, Nepal

## Abstract

**Background:**

Modern therapeutic medicine is historically based on indigenous therapies and ethnopharmacological uses, which have become recognized tools in the search for new sources of pharmaceuticals. Globalization of herbal medicine along with uncontrolled exploitative practices and lack of concerted conservation efforts, have pushed many of Nepal's medicinal plants to the verge of extinction. Sustainable utilization and management of medicinal plants, based on traditional knowledge, is therefore necessary.

**Methods:**

After establishing verbal informed consent with participating communities, five field surveys, roughly 20 days in duration, were carried out. In all, 176 schedules were surveyed, and 52 participants were consulted through focus group discussions and informal meetings. Altogether, 24 key informants were surveyed to verify and validate the data. A total of 252 individuals, representing non-timber forest product (NTFP) collectors, cultivators, traders, traditional healers (*Baidhya*), community members, etc. participated in study. Medicinal plants were free-listed and their vernacular names and folk uses were collected, recorded, and applied to assess agreement among respondents about traditional medicines, markets and management.

**Results:**

Within the study area, medicinal herbs were the main ingredients of traditional therapies, and they were considered a main lifeline and frequently were the first choice. About 55% plants were ethnomedicinal, and about 37% of ethnomedicinal plants possessed the highest informant consensus value (0.86–1.00). Use of *Cordyceps sinensis* as an aphrodisiac, *Berberis asiatica* for eye problems, *Bergenia ciliata* for disintegration of calculi, *Sapindus mukorossi* for dandruff, and *Zanthoxylum armatum* for toothache were the most frequently mentioned. These species possess potential for pharmacology.

**Conclusion:**

Medicinal plants are inseparable from local livelihoods because they have long been collected, consumed, and managed through local customs and knowledge. Management of traditional therapies is urged, because the therapies are empirically and knowledge based, often culturally inherited and important to pharmacology and local livelihoods. However, traditional therapies are currently being eroded due to changing lifestyles, perceptions, social transformations, and acculturation.

## Introduction

Nepal is ranked as 9^th^ among the Asian countries for its floral wealth with an estimated 9,000 species of flowering plants [1]. So far, 6,653 species of flowering plants have been reported [2]. Among these, about 50% fall under the rubrics "useful" [3] and "ethnobotanical" [4], and about 25%–50% are ethnomedicinals [5,6]. Catalogues have recorded 1,792 [7] to 2,331 [8] useful medicinal and aromatic plants in Nepal, reporting their importance in alleviating human suffering because they have long been used for subsistence, home remedies, and traditional therapies [5-8]. These plants are also important for local livelihoods [9] and income generation [10], and they do fetch higher market prices [11].

National and regional demands for herbal medicine are accelerating [12,13], and globalization of herbal medicine, along with uncontrolled exploitative practices and lack of concerted conservation efforts, now threaten the country’s medicinal plants [14,15]. Numerous drugs have been introduced to international markets [16] through validation of traditional medicines [17], indigenous therapies [18,19], and ethnopharmacological practices [20]. Sustainable utilization and management of medicinal plants based on traditional knowledge is therefore necessary. It is imperative that the medicinal plants, their traditional uses, and management practices be catalogued as part of a larger conservation effort toward "rescuing a global heritage" [21]. This effort should acknowledge empirical data and ethnoecological knowledge [22,23]. Far-west Nepal is the least studied area in Nepal [20]. The present study was thus an attempt to catalogue the important medicinal plants of Far-west Nepal along with their traditional uses and management interventions.

### Methodology

#### Study area

Three districts along the western border of Nepal—Baitadi, Darchula, and Dadeldhura—were selected for the present study. The districts extend between 29°01’–30°15’ N latitude and 80°15’–81° 09’ E longitude; their elevation ranges between 257 m and 7132 m above sea level. In Dadeldhura, the following villages were visited: Jogbudha, Rupal, Sirsha and Sadani. In Baitadi, the villages of Siddeswor, Siddnath, Patan, and Pancheswor were visited. In Darchula, the villages of Maikholi, Khar, Khalanga, Rapla, Dumling, and Gokuleswor were visited. Community forests and their user groups were consulted in the villages of Sigas, Rameswor, Madhu, Bhumi Raj and Trishuli (Baitadi district); Tham and Dumling (Darchula district); and Shivsundari, Trishuli Mahila, Parshuram, and Siddnath (Dadeldhura district). National forests were assessed at Jogbudha and Deurali (Dadeldhura district); Pancheswor and Melauli (Baitadi district); and Gokuleswor and Rapla (Darchula district). Local markets and traders were consulted in Amargadhi and Bagarkot (Dadeldhura district); Khalanga and Dumling (Darchula district); and Khodpe, Patan, and Melauli (Baitadi district). Due to variations in altitude, topography, and bio-climate in Far-west Nepal, the diversity of medicinal plants and knowledge of their utilization varied widely.

**Figure 1:**
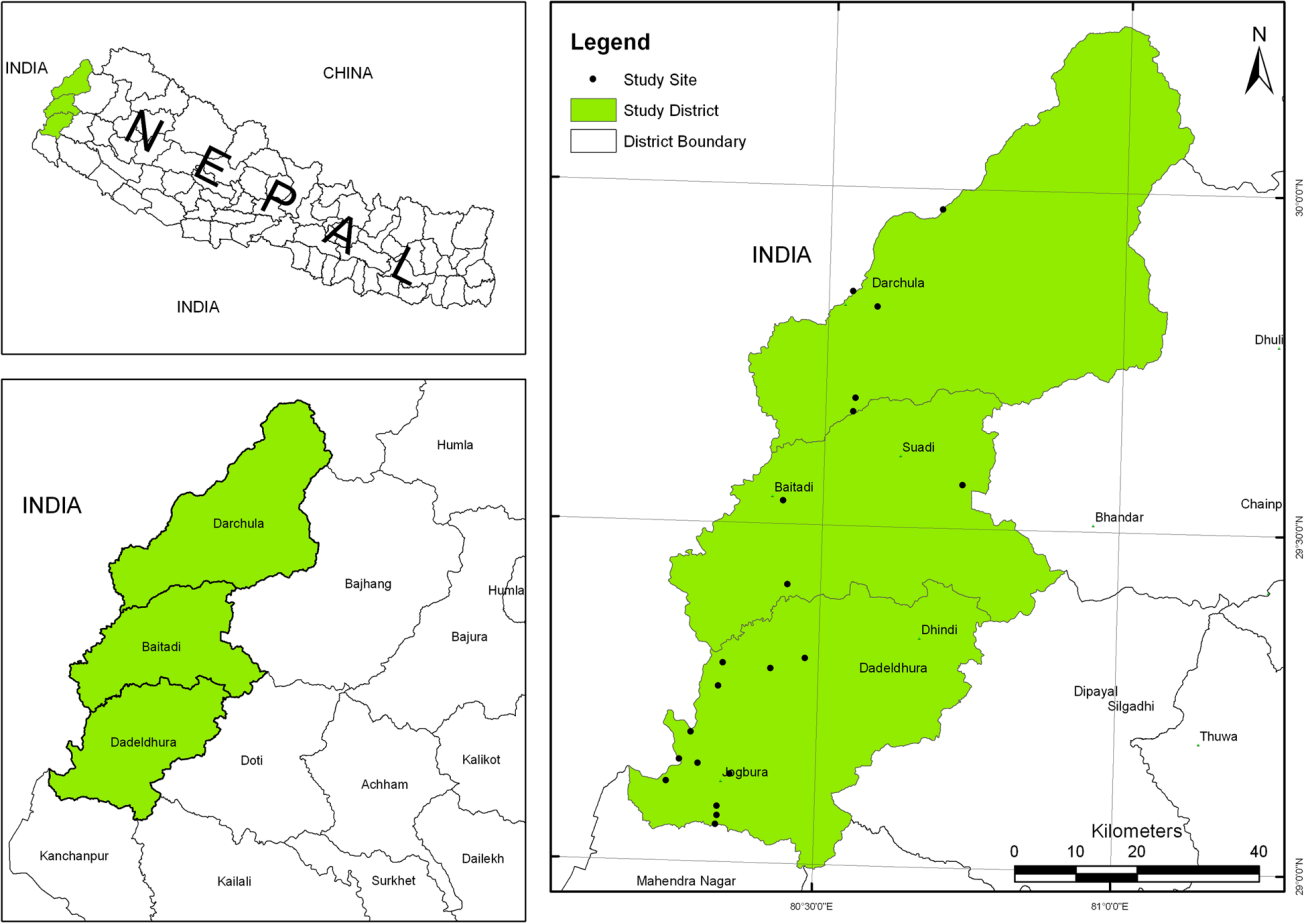
Study area map with details of study sites.

There are a number of ethnic groups in this region, dominated by the Kshetri (more than 57%), Brahmin (about 20%), Kami (6%), Sarki (3%), Mahar (2%), and a few others within the study area. The first two groups are relatively privileged [24], the Kami and Sarki are categorized as *Dalits,* and the Mahar and others are categorized as *Janajati,* based on the socio-cultural and class system of Nepal. The *Dalits* and *Janajati* groups are receiving special easy access opportunities provided by the government (Figure 1).

#### Field surveys and data collection

After establishing verbal informed consent with the participating communities, five field surveys, roughly 20 days in length, were carried out in different seasons: Spring surveys were conducted in May 2006 and March–April 2008; summer surveys were conducted in August 2009; and winter surveys were conducted in December 2006 and February 2007. Field visits were not carried out in Autumn, because it is festival season in Nepal. The primary methods of data collection consisted of group discussions, informal meetings, schedule surveys, and field observations. In all, 176 schedules were surveyed and 52 participants were consulted in focus group discussions and informal meetings. Informal meetings were held in villages during the evening, while team members were staying with local communities. Altogether, 24 key informants were surveyed to verify and validate data and information [25]. A total of 252 individuals, representing NTFP collectors, cultivators, traders, traditional healers (*Baidhya*), community members, etc. participated in the study. In particular, elderly people, forest guards, and women representing different ethnic groups, castes, and occupations were encouraged to participate. Among the participants, 60% were Kshetri, 20% Brahmin, 12% *Janajati* (Chaudhary, Mahar, etc.) and 8% *Dalits* (Chuhar, Bijale, Lawad, Pariyar etc.). Twenty one percent of participants were women. There were altogether 88% respondents from Kshetri, Brahmin, and *Dalits,* and the similar account of ethnic groups was also found in three study districts [26].

All species encountered during participatory field observations were free-listed, and vouchers of medicinal plant species were collected during the day and displayed in the evening for discussions. Most of the species were identified in the field using references [27,28]. Common species that were frequently seen, spot-identified by study team members and local assistants, and well known by their dialect names were used only for discussion; no voucher specimens were collected for further identification. The remaining unidentified species were vouchered and stored in the National Herbarium and Plant Laboratories (KATH), Godawari, Lalitpur, Nepal. The collection of voucher specimens, along with additional information, was facilitated by local assistants. Data and information were sought on the interest of collectors/cultivators, their household economy, and their patterns of use and management of medicinal plants, etc. A wealth index was established, and active involvement of people in medicinal plant cultivation, collection and trade, as well as the net cash income of consecutive two years (2006 and 2007), were analyzed following the literature [29-32]. Vernacular names and folk uses of voucher specimens were collected and used to assess consensus among respondents [33]. Matching information from at least three respondents (mentions) was counted as a common response for analysis. The species were assessed for informant consensus value (ICV), and species with more than 0.85 ICV were considered for further analysis.

## Results and discussion

This study recorded the use of 238 plant species, which were distributed among 209 genera and 92 families. Of these, 132 species (55%) had an ethnomedicinal use. The families Asteraceae, Fabaceae, and Euphorbiaceae, and the genera *Euphorbia, Ficus,* and *Swertia* were significant sources of ingredients for folk remedies. Asteraceae and Fabaceae each contributed seven species, followed by six species from Euphorbiaceae. There were three species used as ethnomedicinals from *Ficus,* and two species used from *Euphorbia, Rhododendron,* and *Swertia*. The ethnomedicinal species were assessed for informant consensus value (ICV). A total of 49 plant species were found to have more than three responses (mentions) and an ICV greater than 0.85 (Table 1), indicating that they are worthy of pharmacological study.

**Table 1:** Diseases/disorders, plant parts in use and mode of preparations

**Category and diseases/disorders**	**Total number of species used**	**Specific disorder and species and its highest mentions and IC value**		
**NVS** - Memory longevity, migraine, epilepsy, laxative, aphrodisiac, tonic, stimulant	19	Aphrodisiac – *Cs* (50) ICV = 1	Epilepsy – *Ng* (31) ICV = 0.97	Laxative – *Pe* (28) ICV = 0.97
**OTH** - Pesticidal, repellant, paralysis, toothache	14	Pesticidal – *Ac* (42) ICV = 0.96	Toothache – *Za* (45) ICV = 0.95	Paralysis – *Ca* (24) ICV = 0.96
**DIG** - Anthelmintic, nausea, vomiting, diarrhea, gallstone, indigestion, dysentery, gastric trouble	44	Gall stone – *Bc* (43) ICV = 0.95	Anthelmintic – *VJ* (32) ICV = 0.95	Nausea/vomiting – *Ct* (34) ICV = 0.95
**OPTH** - Eye disease	4	Eye complaints - *Ba* (34) ICV = 1	Eye complaints – *Pn* (31) ICV = 0.95	Eye complaints – *Ap* (31) ICV = 0.95
**DER** - Scabies, skin diseases, allergy, moles, hair problems	13	Hair problems - *Sam* (34) ICV = 0.93	Scabies – *Cop* (25) ICV = 0.93	Skin promlem – *Pz* (25) ICV = 0.93
**MSK** - Antispasmodic, sprain, fracture, arthritis, rheumatism	10	Arthritis – *Jc* (33) ICV = 0.96	Sprain – *Bv* (32) ICV = 0.96	Fractures – *Ud* (32) ICV = 0.96
**INJ** - Cuts, wounds, sore, inflammation	16	Cuts/wounds - *Dh* (31) ICV = 0.86	Wounds – *Cas* (31) ICV = 0.86	Cuts – *Eph* (31) ICV = 0.86
**GUS** - Urinary complaints, gonorrhea, syphilis, diuretics	11	Diuretics – *Pe* (31) ICV = 0.92	Diuretics – *Ms* (31) ICV = 0.92	Urinary complaints – *Ach* (26) ICV = 0.92
**RES** - Asthma, pneumonia, bronchitis, jaundice, cough/cold	18	Jaundice – *Cr* (27) ICV = 0.91	Pneumonia – *Mh* (24) ICV = 0.91	Asthma – *Ms* (16) ICV = 0.91
**INF** - Burn, scald, boil, earache, fever	16	Fever – *Sc* (26) ICV = 0.90	Burns – *Cn* (19) ICV = 0.90	Boils - *Pr* (19) ICV = 0.90
**PRG** – Lactation, easy delivery	3	Easy Delivery - *Ari* (23) ICV = 0.97	Lactation – *Ar* (21) ICV = 0.97	
**CVC** - Bleeding, blood disorders, liver disorders, spleen disorders, jaundice, hemorrhage	15	Blood disorders – *Mk* (22) ICV = 0.94	Haemorrhage – *Ari* (21) ICV = 0.94	Liver disorder – Mo (15) ICV = 0.94
**POS** - Antidote, snake bite, scorpion sting, fish stupefying	4	Snake bite – *Pp* (18) ICV = 0.90	Scorpion sting – Acv (18) ICV = 0.90	Antidote – Asp (15) ICV = 0.90

**CVC** – Cardiovascular, **DER** – Dermatological, **DIG** – Digestive, **GUS** – Genito-urinal, **INF** – Infections, **INJ** – Injuries, **MSK** – Musculo-skeletal, **NVS** – Nervous, **OPTH** - Ophthalmic, **OTH** – Others, **POS** – Poisonous, **PRG** – Pregnancy, **RES** – Respiratory

*Aa = Angelica archangelica* (KU 07210)*, Ac = Acorus calamus* (KU 07234)*, Ach = Anthocephalus chinensis* (KU 07220)*,Acv = Adiantum capillis-veneris* (KU 07224)*, Ap = Abrus precatorius* (KU 07209)*, Ar = Asparagus racemosus* (KU 07221)*, Ari = Astilbe rivularis* (KU 07231)*, Asp = Aconitum species* (KU 07233)*, Ba = Berberis asiatica* (KU 07246)*,Bc = Bergenia ciliata* (KU 07252)*, Bv = Bauhinea vahlii* (KU 07240)*, Ca = Curcuma aromatica, Cas = Centella asiatica* (KU 07276)*, Cn = Coriaria nepalensis* (KU 07247)*,Cs = Cordyceps sinensis* (KU 07241)*, Co = Curculigo orchioides* (BKU 020)*,Cop = Colebrookea oppositifolia* (KU 07232)*, Cr = Cuscuta reflexa* (BKU 053)*, Ct = Cinnamomum tamala* (BBU 095)*, Dh = Dactylorhiza hatagirea* (KU 07271)*, Ep = Eclipta prostrata* (BKU 055)*, Eph = Entada phaseoloides* (KU 07236)*, Fn = Fragaria nubicola* (KU 07242)*, Jc = Jatropha curcas* (BBU 056)*, Jr = Juglans regia* (KU 07272)*, Mc = Morchella conica* (KU 07243)*, Mh = Melothria heterophylla* (KU 07255)*, Mk = Murraya koenigii* (BBU 091)*, Mo = Moringa oleifera* (KU 07237)*, Mp = Mallotus philippensis* (BKU 092)*, Ms = Mentha spicata* (BKU 058)*, Ng = Nardostachys grandiflora* (DBU 060)*, Ns = Neopicrorhiza scrophulariflora* (DKU 090)*,*

*Osp = Osmanthus species* (KU 07244)*, Ow = Osyris wightiana* (KU 07250)*, Pe = Phyllanthus emblica* (BKU 135)*, Pn = Parnasia nubicola* (KU 205)*, Pp = Paris polyphylla* (DBU 134)*, Pr = Pinus roxburghii, Pu = Prinsepia utilis* (BKU 136)*, Pz = Plumbago zeylanica* (BBU 089)*, Sam = Sapindus mukorossi* (DKU 087)*, Sc = Swertia chirayita* (DBU 067)*, Sd = Scutellaria discolor* (KU 07263)*, Sm = Sophora mollis* (KU 07275)*, Tc = Thalictrum cultarum* (KU 07284)*, Ud = Urtica dioica* (KU 07290)*, Vj = Valeriana jatamansii* (KU 07279)*, Za = Zanthoxylum armatum* (KU 07291).

### Collection of medicinal plants

Community forests were the main sources of collection of medicinal plant products. We recorded that 49.5% collections were gathered from community forests and 18.5% from government-managed forests. The latter have been exploited as open-access free commodities, therefore their contribution to furnishing provisional products to local communities has been gradually declining. We found that only about 33% of the collections were used for subsistence (primarily as ingredient for folk remedies and food). The rest (about 67%) was sold, thanks to increasing market prices of the wild crafted products. Collection for the market was the predominant reason behind collection in Nepal [34] and India [35]. Marketing of medicinal plant products is important to address the need for household commodities; the study area is rural and distant from road transport.

Since forests of the Himalaya are the richest habitat for medicinal plants [36] and a common property resource in Nepal [37], *Dalits* and *Janajati* groups within the study area were frequent collectors, and they gathered a disproportionate amount of forest products—particularly medicinal plants to address their subsistence and accessory needs. The overall increasing trend in collection and consumption was 16%. The increase in the number of collectors per annum was about 10% for the Brahmin and Kshetri, 35% for the *Janajati,* and 40% for the *Dalits.* The trend was consistent with their dependency, as the *Dalits* are more dependent on forests [38]. Such increases were attributed to the expansion of commercial demand for medicinal plants and higher market prices.

All family members of local farmers and traditional healers were generalist collectors, but their children and elders were sometimes used in collection of forest products from nearby and accessible areas. Most household heads that have been collecting plants/products since infancy use their children for support. We found that about 20% of the collectors were children, but their involvement was limited, particularly to private farmlands, lower elevations, and accessible areas. Children were guided to collect produce and products of *Prinsepia utilis, Rhus parviflora, Murraya koenigii*, *Zanthoxylum armatum, Berberis asiatica, Centella asiatica,* etc. In particular, children were trained to collect the leaves and fruits of useful species. Children and elders were most seldom used in the collection of high-value medicinal plants and products found at higher elevations, such as *Cordyceps sinensis, Morchella species,* etc., thanks to the climatic and physiographic adversity associated with their collection. The species valued for their rhizomes and bark were commonly uprooted or debarked by elderly groups. There was a range of collection of medicinal plants in each household (N = 252) from 0.6 Kg to 720 Kg of fresh products.

Besides forests, private farmlands were also important in the supply of medicinal plant products. About 32% of collections of medicinal plant products were from private farmlands. In some private farmlands, plants such as *Sapindus mukorossi, Z. armatum, Phyllanthus emblica,* and *Swertia chirayita*, which fetch good market prices, were intentionally cultivated. Other medicinal plants that occur naturally in farmlands were *Pyrus pashia, Ficus hispida, Acmella calva, Amaranthus viridus, Choerospondias axillaris,* etc. These plants were under-utilized [39].

### Marketing of medicinal plants

As shown by Olsen and Larsen [20], the livelihoods of people of western Nepal heavily depend on the collection and trade of medicinal plants. Within the three districts that were studied, a total of 42 species or products were found to be collected and traded for both subsistence and commercial purposes (Table 2). The data ranged from 40 for Baitadi to 36 for Darchula and 28 for Dadeldhura. Twenty two items were common to all three districts. Earlier reports indicated that the number of traded species in and around the study area was between 26 and 42 [31,32]. The numbers for Nepal as a whole are between 125 and 178 [40-42].

**Table 2:** List of species/products traded from districts

**SN**	**Trade name**	***Botanical name***	**Baitadi**	**Dadeldhura**	**Darchula**	**Common in all districts**
1.	Amala	*Phyllanthus emblica*	+	+	+	+
2.	Bhorla Bokra	*Bauhinia vahlii*	+	+	+	+
3.	Bhyakur	*Dioscorea species*	+	+	+	+
4.	Bishjara	*Aconitum species*	+	+	+	+
5.	Chiraito	*Swertia chirayita*	+	+	+	+
6.	Chutro	*Berberia asiatica*	+	+	+	+
7.	Dalchini	*Cinnamomum tamala*	+	+	+	+
8.	Daruhaldi	*Mahonia nepalensis*	+	+	+	+
9.	Jhyau	*Lichen species*	+	+	+	+
10.	Kachur	*Curcuma aromatica*	+	+	+	+
11.	Kauloko bokra	*Persea species*	+	+	+	+
12.	Miscellaneous		+	+	+	+
13.	Pakhanbed	*Bergenia ciliata*	+	+	+	+
14.	Pawan Bokra		+	+	+	+
15.	Rittha	*Sapindus mukorossi*	+	+	+	+
16.	Tejpat	*Cinnamomum tamala*	+	+	+	+
17.	Timur	*Zanthoxylum armatum*	+	+	+	+
18.	Chatiwan	*Alstonia scholaris*	+	+	+	+
19.	Chyau	*Mushroom*	+	+	+	+
20.	Kakarsingi	*Pistachia chinensis*	+	+	+	+
21.	Satuwa	*Paris polyphylla*	+	+	+	+
22.	Kurilo	*Asparagus racemosus*	+	+	+	+
23.	Barro	*Terminalia belerica*	+	+		
24.	Bojho	*Acorus calamus*	+	+		
25.	Jiwanti	*Ephemerantha macarei*	+	+		
26.	Lokta	*Daphne bholua*	+	+		
27.	Somlata	*Ephedra gerardiana*	+	+		
28.	Sugandhwal	*Valeriana wallichii*	+	+		
29.	Bajradanti	*Potentilla fruticosa*	+		+	
30.	Bhojpatra	*Betula utilis*	+		+	
31.	Bhutkesh	*Jurenia dolomiea*	+		+	
32.	Dhupipat	*Juniperus species*	+		+	
33.	Guchhi	*Morchella conica*	+		+	
34.	Katush	*Castanopsis species*	+		+	
35.	Kutjara		+		+	
36.	Kutki	*Neopicrorhiza scrohpularoflora*	+		+	
37.	Nirmansi	*Delphinium denundatum*	+		+	
38.	Setak chini	*Moringa oleifera*	+		+	
39.	Siltimur	*Litsea cubeba*	+		+	
40.	Thingresalla	*Abies spetabilis*	+		+	
41.	Aank bhuwa	*Calotropis gigantea*			+	
42.	Yarsagumba	*Cordyceps sinensis*			+	

An average of 20,000 tons of raw materials, worth between $8.6–$27 million ($US) annually, are traded and/or exported from Nepal [43,44]. However, official records only record a much lower volume (25%–50%). Of the five political divisions in Nepal (East, Central, West, Mid-west and Far-west), the Far-west contributes about 20% of the total volume of medicinal and aromatic plants traded from country. The three study districts (Baitadi, Dadeldhura, and Darchula) alone contribute 17.4% of the country’s total traded volume; this accounts for 78% of the Far-west trade [45]. Among the three study districts, Baitadi is rich in medicinal and aromatic plants and alone contributes about 10% of the national traded volume. *S. mukorossi* and *Z. armatum* were the species traded in the largest volume in the country, and both the species are indigenous to western Nepal.

Income from medicinal plants comprised a significant portion (20%) of the annual cash income of local people in study area. It was computed against different wealth indices, and it represented the highest percentage of income for the *Janajati* and *Dalits* groups, which was consistent with their gradual increase in numbers per annum. The annual increase in income from medicinal plant collection and cultivation was shown to go from N.Rs. 4,223 in 2006 to N.Rs. 5,802 in 2007. A similar increase was also noted by Acharya and Tamrakar [30] from N.Rs. 1,326 in 2005 to N.Rs. 9,290 in 2009. In other parts of Nepal, the contribution of medicinal plants to annual cash income was about 15% [43,44], and in some cases was as high as 30%–50% [18].

### Traditional use

A total of 49 species possessed an ICV more than 0.85, and all these species possessed matching information from at least 20 respondents (mentions). Use of plants for treatment of 60 ailments in 13 disease categories was recorded following Phillips and Gentry [46] (Table 1). Digestive system disorders were frequent and their treatment with locally available medicinal plants was the most consistent with the findings of Kumar *et al.*[47]. Informants described the usefulness of 44 species for digestive disorders (Table 1). There was a broad consensus for the use of *C. sinensis* as an aphrodisiac and *B. asiatica* for eye problems. Furthermore, *Bergenia ciliata* was often mentioned for the treatment of the disintegration of calculi, *S. mukorossi* for dandruff, and *Z. armatum* for toothache; these treatments share the highest consensus value. This high ICV indicates that use of these species is widely known in study area, and most of the respondents concurred regarding the particular usage. This paper takes the position that informant consensus is a reliable method of selecting medicinal plant for pharmacological evaluation [48].

Traditional uses of *C. sinensis* as a tonic, aphrodisiac, immuno-stimulant, and for retention of memory coincided with previous pharmacological findings. The adenosine found in *C. sinensis* has widespread effects on circulation of blood, cerebral and coronary effects [49,50], prevention of cardiac arrhythmias [51], inhibition of neurotransmitter release [52], potentiating immune system [53], and antitumor activity [54]. The Ayurvedic and folk usage of *B. ciliata* as an antidiarrheal and antidysenteric [55,56] also corroborated earlier reports [6,9]. The fruits of *Z. armatum* were commonly used to treat toothache in folklore and Ayurvedic medicines, and their anti-inflammatory [57] and antibacterial [58] actions have been substantiated. The use of root bark extract of *B. asiatica* to treat conjunctivitis was common [55,59], and its effectiveness as an antimicrobial [60], anesthetic, antihypertensive, and pigment inducer [61] has been clinically verified. *Sapindus* fruits were commonly applied to fight dandruff. *Sapindus’* emetic and expectorant properties have previously been mentioned in the Ayurveda [55] and antichlamydiac property in pharmacology [62]. The folk use of *Cuscuta reflexa* for jaundice was supported by pharmacological studies, showing it possesses strong antibacterial actions [63].

### Plant parts used and mode of preparation

**Figure 2:**
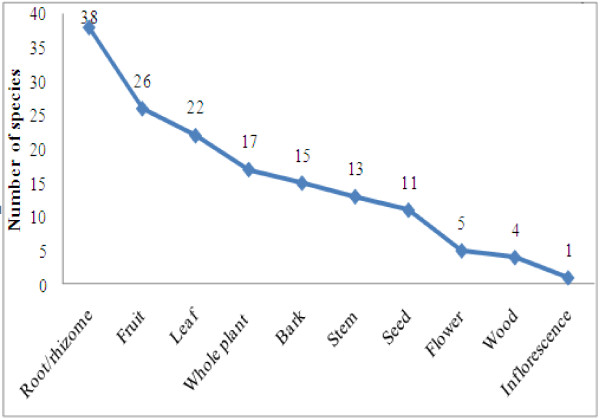
Plants parts used for curving.

**Figure 3:**
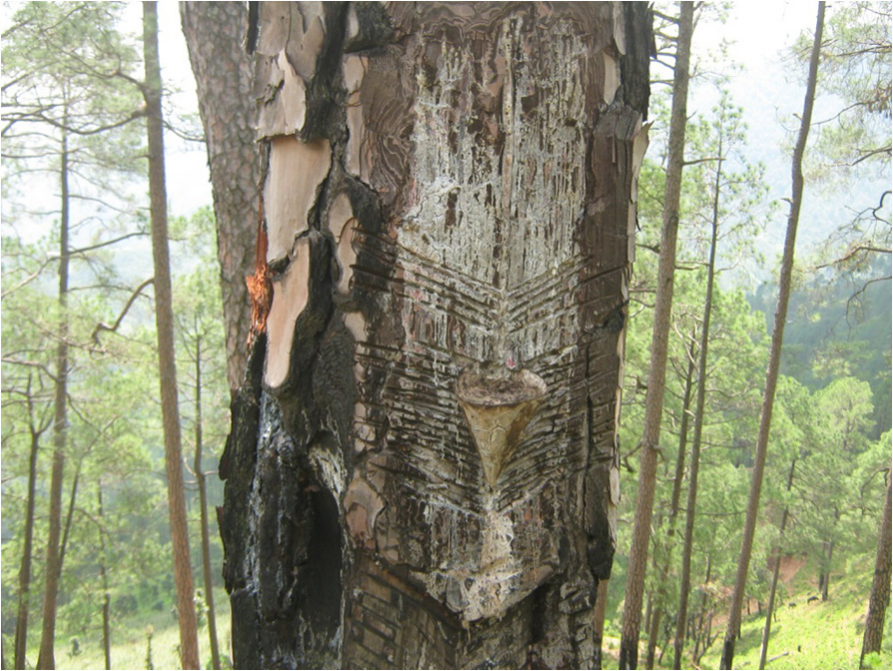
Collecting pine resin: useful in boils.

**Figure 4:**
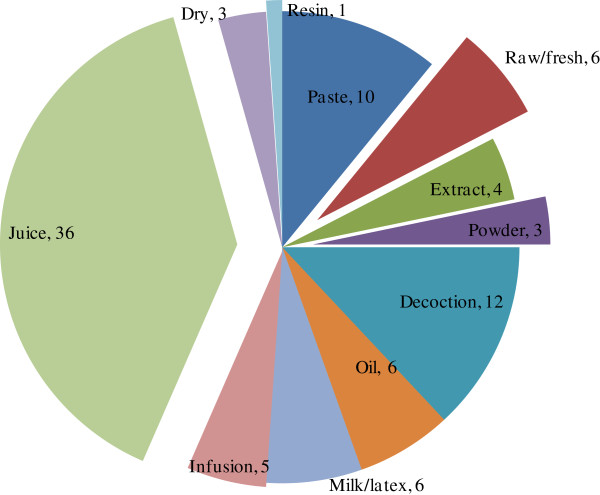
Different modes of preparation use for therapies.

Plant parts used for ethnomedicinal preparations were bark, fruit, flower, inflorescence, leaf, root, rhizome, stem, seed, wood, and the whole plant. The most frequently utilized plant parts were roots and rhizomes of 38 species, followed by fruits of 26 species, leaves of 22 species, etc. (Figure 2). Underground parts were frequently used, and this was attributed to presence of bioactive compounds [64]. Preparation methods for therapies included decoction, drying, and extraction. Plant infusion/smoke, juice, latex, oil, paste, powder, raw/fresh and resin were also applied (Figure 3). Plant juice (39.13%) was most commonly used, followed by decoction (13.04%), paste (10.86%), etc. The most popular forms of medicinal preparations in western Nepal are juice, decoction, paste, infusion, and powder [65] (Figure 4).

### Traditional therapies

Home herbal remedies, folklore medicine, and the *Baidhya* healing system are commonly found in the study area. The persistence of these traditional therapies is due to the high cost of modern medicine and the absence of trained physicians. It is estimated that there is one physician per 30,000–100,000 people in Nepal [66], whereas there is at least one healer for every 100 people [67]. Data show that there is at least one health worker for every 670 people in Baitadi, 663 in Dadeldhura, and 509 in Darchula district [68]. Interest in traditional therapies has gradually increased over the recent years [6,39,69]. All these reasons foster the persistence of traditional therapies in the study area. This is reinforced by the preference of local people for traditional medicine, thanks to their belief in its effectiveness and to a lack of alternative choices [70,71]. All these therapies were culturally intertwined. The number of plants used as ethnomedicines was also higher in the mountains of the Nepalese Himalayas [5]. However, traditional knowledge and the plants for folk therapies are gradually being endangered due to changing lifestyles, perceptions, social transformations, and acculturation.

### Management

The many uses of medicinal plants for trade and subsistence needs have led to conflict and overexploitation. An account of the multiple uses of medicinal plants that in turn threaten those very plants as a result of overharvesting has been noted in India [72]. Deliberate forest fires and premature harvests endanger existing populations of medicinal plants. The quantity and quality of medicinal plants within the study area have been degraded by market-led premature exploitation and climate change. The changes to plant phenology, range, and growth behavior as a result of climate change also jeopardize species survival and quality products [73,74]. Price differentials between wild and cultivated products, which reflect the demand for wild crafted products, encourage unsustainable collection. Over-harvesting was also threat to many indigenous medicinal plant species, such as *Z. armatum, N. grandiflora, A. rivularis* etc. The collection of medicinal plants in a sustainable manner is imperative for development and conservation [75].

**Figure 5:**
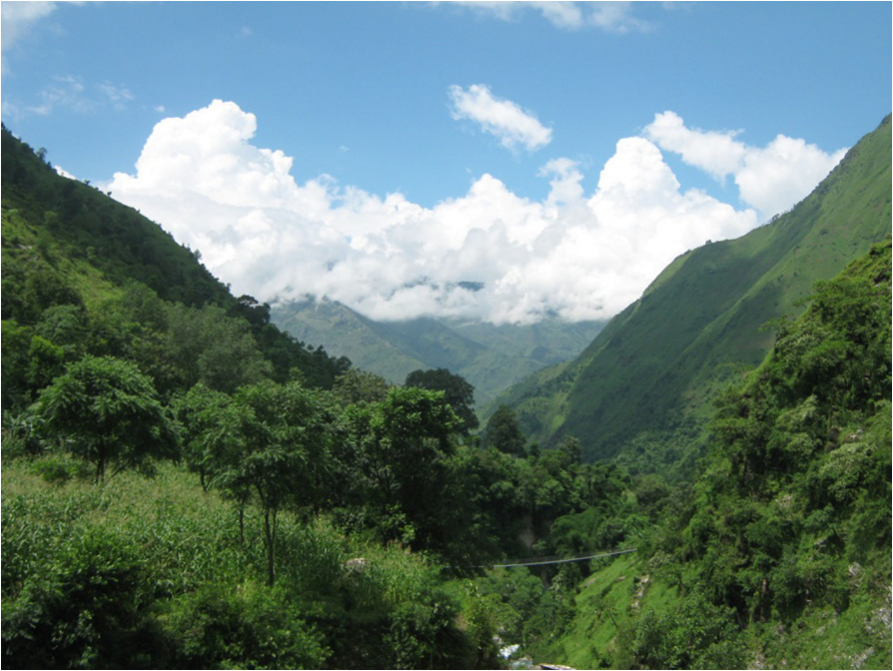
Ecological landscape favorable for medicinal plant growth.

In order to conserve indigenous and high value medicinal plants, the government of Nepal outlawed the collection and trade of 17 important medicinal plant species [76] and has urged citizens to cultivate 30 highly valued species [77]. These initiatives have proven inadequate [78]. Because of the firm relationship between medicinal plants and rural livelihood (Figure 5), it is unlikely that a legal ban can be enforced [79] due to complex management systems [80], invigorating cultivation, and sustainable management. Community-based organizations have also attempted to conserve medicinal plant resources and revitalize indigenous resource management systems. The Himalayan Amchi Association (HAA), an institution aimed at safeguarding traditional health care knowledge [81], is devoted to protect medicinal plants and strengthen the knowledge of *Amchi* healers [82,83]. The *Amchi* health care system, influenced from Tibetan Chinese medicine, is active in the high hills and mountainous districts of Nepal [7]. The *Baidhya* healing system, prevalent in the mid-hills of western Nepal [14], is influenced by the *Ayurveda*. *Baidhya* medicinal practitioners (particularly of western Nepal mid hills [71] and adjoining areas of India [84]) were common among privileged groups and pursued remedies to cure diseases and aliments by using nearby medicinal plants. Their knowledge base for therapies stems from custom, livelihood strategies, and available resources. Because of their prolonged existence and use, these systems and therapies are inseparable from local culture. Strengthening wise use and conservation of resources and knowledge of healing systems, which are culturally inherited and valued as well as scientifically important, may complement pharmacology [19] and the livelihood of communities inhabiting remote and high-altitude areas of the western Himalaya [85].

## Conclusions

Medicinal plants play a substantial role in the life support systems of local communities of Far-west Nepal. With increasing acceptance and use of medicinal plants in traditional therapies, and with increasing commercial demands over the years, the consumption and collection of medicinal plants is accelerating and thus endangering the extant populations. Over-harvesting threatens many indigenous medicinal plant species, such as *Z. armatum, N. grandiflora, A. rivularis* etc. Collection and consumption of medicinal plants in a sustainable manner is an integrated process with potential for development and conservation. Indigenous knowledge of medicinal plant use for traditional therapies and sustainable management in Nepal is empirically based, culturally inherited and intertwined, and important to pharmacology and local livelihoods. However, it is currently being eroded due to changing lifestyles, perceptions, social transformations, and acculturation. The conservation and sound management of indigenous knowledge of medicinal plants is thus strongly urged.

## Competing interests

The authors declare that they have no competing interests.

## Authors’ contributions

RMK, RPA, and LM carried out field studies. RMK and RPA analyzed data and prepared manuscript. RWB edited the manuscript, further analyzed the data, and finalized the article. All authors approved the final version of this manuscript.

## References

[B1] Bhattarai KR Maren IE Chaudhary RP Medicinal plant knowledge of the Panchase region in the middle hills of the Nepalese Himalaya Banko Janakari 2011 21 2 31 39

[B2] Kunwar RM Shrestha K Dhungana SK Shrestha PR Shrestha KK Floral Biodiversity of Nepal: an update Journal of Natural History Museum 2010 25 295 311

[B3] Uprety Y Asselin H Boon EK Yadav S Shrestha KK Indigenous use and bio-efficacy of medicinal plants in the Rasuwa District, Central Nepal J Ethnobiol Ethnomed 2010 6 3 http://www.ethnobiomed.com/content/6/1/3 10.1186/1746-4269-6-3 20102631 PMC2823594

[B4] Kunwar RM Bussmann RW Ethnobotany in the Nepal Himalaya J Ethnobiol Ethnomed 2008 4 24 http://www.ethnobiomed.com/content/4/1/24 10.1186/1746-4269-4-24 19055723 PMC2639547

[B5] Manandhar NP Plants and People of Nepal 2002 Oregon, USA: Timber press Inc. Portland 599

[B6] Kunwar RM Nepal BK Kshhetri HB Rai SK Bussmann RW Ethnomedicine in the Himalaya: a case study from Dolpa, Humla, Jumla and Mustang district of Nepal J Ethnobiol Ethnomed 2006 2 27 http://www.ethnobiomed.com/content/2/1/27 10.1186/1746-4269-2-27 16749924 PMC1513199

[B7] Rokaya MB Munzbergova Z Shrestha MR Timsina B Ethnobotanical study of medicinal plants from the Humla district of western Nepal J Ethnopharmacol 2010 130 485 504 10.1016/j.jep.2010.05.036 20553834

[B8] Baral SR Kurmi PP Compendium of medicinal plants in Nepal 2006 Chabahil, Kathmandu, Nepal: Rachana Sharma publishers 534

[B9] Shengji JP Ethnoecological approaches of traditional medicine studies: some experiences from Asia Pharmacoceutical Biology 2001 39 74 79 10.1076/phbi.39.s1.74.0005 21554174

[B10] WHO WHO traditional medicine strategy 2002–2005 2002 Geneva: World Health Organization

[B11] Tiwari S Robinson J Amatya G Bhattarai NK, Karki M Community based approaches to conservation and management of MAPs for sustainable livelihood in Doti district, West Nepal Proceeding of local experience-based national strategy for organic production and management of MAPs/NTFPs in Nepal 2004 Kathmandu: IDRC, MAPPA and Government of Nepal 97 127

[B12] WWF Medicinal plant trade: wildlife trade 2000 World Wildlife Foundation http://wwf.panda.org/who_we_are/wwf_offices/india/?uProjectID=9Z0718

[B13] Karki M Thomas YA, Karki M, Gurung K, Parajuli D The organic production of medicinal and aromatic plants: a strategy for improved value-addition and marketing of products from the Himalayas Proceeding of wise practices and experimental learning in conservation and management of Himalayan medicinal plants 2005 Kathmandu, Nepal: Ministry of Forests and Soil Conservation 56 69

[B14] Singh MP Malla SB Rajbhandary SB Manandhar A Medicinal plants of Nepal – retrospects and prospects Economic Botany 1979 33 185 198 10.1007/BF02858287

[B15] Olsen CS Larsen HO Alpine medicinal plant trade and Himalayan mountain livelihood strategies The Geographical Journal 2003 169 243 254 10.1111/1475-4959.00088

[B16] Sharma PP Mujundar AM Traditional knowledge on plants from Toranmal Plateau of Maharastra, India Indian Journal of Traditional Knowledge 2003 2 292 296

[B17] Bussmann RW Ambasht RS, Ambasht NK Ethnobotany and biodiversity conservation Modern trends in applied terrestrial ecology 2002 Kluwer Publishers: New York 345 362

[B18] Bhattarai NK Biodiversity-people interface in Nepal, Medicinal Plants for Forest Conservation and Health Care 1997 FAO and UN Rome, Italy: NWFP Bulletin 11

[B19] Patwardhan B Warude D Pushpangadan P Bhatt N Ayurveda and traditional Chinese medicine: a comparative overview eCAM 2005 2 4 465 473 16322803 10.1093/ecam/neh140 PMC1297513

[B20] Kunwar RM Uprety Y Burlakoti C Chowdhary CL Bussmann RW Indigenous use and ethnopharmacology of medicinal plants in far-west Nepal Journal of Ethnobotany Research & Applications 2009 7 5 2823562803

[B21] Lambert J Srivastava J Vietmeyer N Medicinal Plants: Rescuing a Global Heritage 1997 USA: The World Bank

[B22] Ramakrishnan PS Chandrashekara UM Elouard C Guilmoto CJ Maikhuri RK Rao KS Sankar S Saxena KG Mountain biodiversity, land use dynamics, and traditional ecological knowledge 2000 New Delhi, India: Oxford and IBH Publishing Co

[B23] Ghimire SK McKey D Thomas YA Himalayan medicinal plant diversity in an ecologically complex high altitude anthropogenic landscape, Dolpo, Nepal Environmental Conservation 2006 33 128 140

[B24] Bennet L Gender, caste and ethnic exclusion in Nepal: following the policy process from analysis to action 2005 Arusha, Tanzania: Paper presented at the conference New Frontiers of Social policy: development in a globalizing world http://siteresources.worldbank.org/INTRANETSOCIALDEVELOPMENT/Resources/Bennett.rev.pdf

[B25] Martin GJ Ethnobotany: A methods manual 2004 London: Earthscan Publications

[B26] Dahal DR Social composition of the population: case/ethnicity and religion in Nepal Population monograph of Nepal 1 Central Bureau of Statistics, Government of Nepal http://cbs.gov.np/wp-content/uploads/2012/Population/Monograph

[B27] Polunin O Stainton A Flowers of the Himalaya 1984 New Delhi-India: Oxford University Press 580

[B28] Stainton A Flowers of the Himalaya, a supplement 1998 New Delhi-India: Oxford University Press 86

[B29] Kunwar RM Ecology and economy of medicinal, aromatic and dye plants for sustainable livelihood, west Nepal: an assessment 2007 Kathmandu, Nepal: Canadian Center for International Studies and Cooperation 32

[B30] Acharya RP Tamrakar R Livelihood and poverty impacts of MAPPA project in west Nepal 2009 Kathmandu, Nepal: International Center for Integrated Mountain Development (ICIMOD) 68

[B31] ANSAB Market Assessment of Non Timber Forest Products in Darchula and Baitadi Districts, Far-west Nepal 2003 Kathmandu: Asia Network for Sustainable Agriculture and Bioresources

[B32] CMAPSL Conservation of medicinal and aromatic plants for sustainable livelihood project. Report 2005 Kathmandu, Nepal: Canadian Center for International Studies and Cooperation

[B33] Trotter RT Logan MH Ektin NL Informant consensus: a new approach for identifying potentially effective medicinal plants Plants in indigenous medicine and diet 1986 Bedford, US: Redgrave Publication Co 91 112

[B34] Kunwar RM Duwadee NPS Ethnobotanical notes on flora of Khaptad National Park, far-western Nepal Himalayan Journal of Sciences 2003 1 25 30

[B35] Narendran K Murthy IK Suresh HS Dattaraja HS Rabindranath NH Sukumar R NTFP extraction, utilization and valuation; a case study from the Nilgiri Biosphere Reserve, India Economic Botany 2001 55 528 538 10.1007/BF02871715

[B36] Ives JD The theory of Himalayan degradation: its validity and application challenged Mountain Research and Development 1987 7 189 199 10.2307/3673192

[B37] Ostrom E Governing and managing forests and other common property resources in a period of climate change 2010 http://www.icimod.org/?q=719

[B38] Gaire D Effectiveness and representation of poor, women and Dalitss in executive committee: process and achievement; a study from the buffer zone of Bardia National Park, Nepal. MSc thesis 2007 Kathmandu, Nepal: Institute of Forestry, Tribhuvan University

[B39] Kunwar RM Mahat L Sharma LN Shrestha KP Kominee H Bussmann RW Underutilized plant species in far west Nepal Journal of Mountain Science 2012 9 589 600 10.1007/s11629-012-2315-8

[B40] Kunwar RM Shrestha KP Bussmann RW Traditional herbal medicine in Far-west Nepal: a pharmacological appraisal J Ethnobiol Ethnomed 2010 6 35 http://www.ethnobiomed.com/content/6/1/35 10.1186/1746-4269-6-35 21144003 PMC3012020

[B41] Bhattarai KR Ghimire M Commercially important medicinal and aromatic plants of Nepal and their distribution pattern and conservation measure along the elevation gradient of the Himalayas Banko Janakari 2006 16 1 3 13

[B42] Srivastava D Resources of Nepalese medicinal and aromatic plants: status and development Plant Resources 2009 31 127 131

[B43] Edward DM Non-Timber Forest Products from Nepal: Aspect of the trade in medicinal and aromatic Plants 1996 Kathmandu, Nepal: Ministry of Forests and Soil Conservation 1 96 FORESC monograph

[B44] Subedi BP Utilization of Non-Timber Forest Products: Issue and strategies for environmental conservation and economic development 1997 Kathmandu, Nepal: Asia Network for Sustainable Agriculture and Bio-resources

[B45] GoN Hamro Ban (Our Forests) 2005 Babarmahal, Kathmandu, Nepal: Government of Nepal. Ministry of Forests and Soil Conservation

[B46] Phillips O Gentry AH The useful plants of Tambopata, Peru: II. Additional hypothesis testing in quantitative ethnobotany Economic Botany 1993 47 33 43 10.1007/BF02862204

[B47] Kumar M Sheikh MA Bussmann RW Ethnomedicinal and ecological status of plants in Garhwal Himalaya, India J Ethnobiol Ethnomed 2011 7 32 http://www.ethnobiomed.com/content/7/1/32 10.1186/1746-4269-7-32 22011477 PMC3212913

[B48] Etuk EU Mohammed BJ Informant consensus selection method: A reliability assessment on medicinal plants used in north western Nigeria for the treatment of diabetes mellitus African Journal of Pharmacy and Pharmacology 2009 3 10 496 500 http://www.academicjournals.org/ajpp/abstracts/abstracts/abstracts2009/October/Etuk%20amd%20Mohammed.htm

[B49] Berne RM The role of adenosine in the regulation of coronary blood flow Cancer Research 1980 47 807 813 10.1161/01.res.47.6.807 6254686

[B50] Toda S Kumura M Ohnishi M Effects of phenolcarboxylic acids on superoxide anion and lipid peroxidation induced by superoxide anion Planta Medica 1991 57 8 10 10.1055/s-2006-960005 1648246

[B51] Pelleg A Porter RS The pharmacology of adenosine Pharmacotherapy 1990 10 157 174 2196534

[B52] Ribeiro JA Purinergic inhibition of neurotransmitter release in the central nervous system Pharmacol Toxicol 1995 77 299 305 10.1111/j.1600-0773.1995.tb01031.x 8778740

[B53] Xu RH Peng XE Chen GZ Chen GL Effects of *Cordyceps sinensis* on natural killer activity and colony formation of B16 melanoma Chin Med J 1992 105 97 101 1597083

[B54] Chen YJ Shiao MS Lee SS Wang SY Effect of *Cordyceps sinensis* on the proliferation and differentiation of human leukemic U937 cells Life Sciences 1997 60 2349 2359 10.1016/S0024-3205(97)00291-9 9194691

[B55] Bajracharya MB Ayurvedic medicinal plant and general treatments 1979 Kathmandu, Nepal: Jore Ganesh Press Pvt. Ltd 230

[B56] Dey AC Indian medicinal plants used in Ayurvedic preparation 1998 Dehra Dun, India: Bishen Singh Mahendra Pal Singh

[B57] Kumar S Ziereis K Wiegrebe W Muller K Medicinal plants from Nepal: evaluation as inhibitors of leukotriene biosynthesis J Ethnopharmacol 2000 70 191 195 10.1016/S0378-8741(00)00203-8 10837982

[B58] Taylor RSL Shahi S Chaudhary RP Chaudhary RP, Subedi BP, Vetaas OR, Aase TH Ethnobotanical research in the proposed Tinjure-Milke-Jaljale Rhododendron conservation area, Nepal Vegetation and society: Their interaction in the Himalayas 2002 Norway: Tribhuvan University, Nepal and University of Bergen 26 37

[B59] Das B Gupta K Materia medica of Ayurveda based on Mandanapala’s Nighantu 1994 New Delhi, India: B. Jain Publishers 780

[B60] Iwasa K Namba H Lee DU Kang SI Structure–activity relationships of protoberberines having antimicrobial activity Planta Medica 1998 64 748 751 10.1055/s-2006-957572 9933992

[B61] Sabir M Bhide MK Study of some pharmacological activities of berberine Indian Journal of Physiology and Pharmacy 1971 15 111 132 4109503

[B62] Garg A Effect of *Calotropis procera* (Ait.) R. Br. flower extract on testicular function of the Indian desert male gerbil *Meriones hurrianae* Jerdon: a biochemical and histological study Indian Journal of Experimental Biology 1979 17 859 862 544463

[B63] Tiwari BR Raut B Screening of selected medicinal plants of Pokhara valley for their antimicrobial activities Journal of Natural History Museum 2009 24 16 20

[B64] Moore PD Trials in Bad Taste Nature 1994 370 410 411

[B65] Burlakoti C Kunwar RM Jha PK, Karmacharya SB, Chettri MK, Thapa CB, Shrestha BB Folk herbal medicines of Mahakali watershed area, Farwest Nepal Medicinal plants in Nepal: An anthology of contemporary research 2008 Kathmandu, Nepal: Ecological Society 187 193

[B66] Gillam S The traditional healer as village health worker Journal of Institute of Medicine 1989 11 67 76

[B67] WRI World Resources – 2005. The wealth of the poor: managing ecosystems to fight poverty 2005 World Resource Institute, USA http://www.wri.org/publication/world-resources-2005-wealth-poor-managing-ecosystems-fight-poverty, http://pdf.wri.org/wrr05_dt_all.pdf

[B68] Anonymous District profile 2010 Kathmandu, Nepal: Mega Research Center and Publication

[B69] Craker LE Gardner ZE Bogers J, Craker LE, Lange D Medicinal plants and tomorrow’s pharmacy Medicinal and aromatic plants. Proc. Frontis Workshop on Medicinal and Aromatic Plants 2006 Wageningen: Nucleus for Strategic Expertise Wageningen University and Research Centre 29 41 Wageningen, The Netherlands, 17–20, April 2005

[B70] Bhattarai NK Traditional herbal medicines used to treat wounds and injuries in Nepal Tropical Doctor 1997 27 1 43 47 9204725 10.1177/00494755970270S114

[B71] Bhattarai NK Medical ethnobotany in the Karnali Zone, Nepal Economic Botany 1992 45 3 257 261

[B72] Appasamy PP Role of NTFPs in subsistence economy: The case of joint forestry project in India Economic Botany 1993 47 3 258 267 10.1007/BF02862292

[B73] Sala O Global biodiversity scenarios for the year 2100 Science 2000 287 1770 1774 10.1126/science.287.5459.1770 10710299

[B74] Kunwar RM Katuwal Y Shrestha RD Karki J Shrestha K Bussmann RW Climate Change, Medicinal Plants and Ethnobotany: Observations and Review Proceeding of first national youth conference on environment 2010 Kathmandu, Nepal: Himalayan Alliance for Climate Change 180 189 http://www.himcca.org

[B75] Hall P Bawa KS Methods to assess the impact of extraction of non-timber forest products on plant populations Economic Botany 1993 47 234 247 10.1007/BF02862289

[B76] GoN Protected Plants of Nepal 2001 Kathmandu, Nepal: Ministry of Forests and Soil Conservation, Forest regulation 1995 and its amendments

[B77] HNCC Prioritized medicinal plants for economic growth in Nepal 2006 Kathmandu, Nepal: Herbs and NTFPs Co-ordination Committee, Department of Plant Resources 125

[B78] Singh AG Kumar A Tewari DD An ethnobotanical survey of medicinal plants used in Terai forest of western Nepal J Ethnobiol Ethnomed 2012 8 19 http://www.ethnobiomed.com/content/8/1/19 10.1186/1746-4269-8-19 22591592 PMC3473258

[B79] Shrestha BB Jha PK Habitat range of two alpine medicinal plants in a trans-Himalayan dry valley, central Nepal Journal of Mountain Science 2009 6 66 77 10.1007/s11629-009-0209-1

[B80] Messerschmidt DA Rayamajhi S Upper slopes forest management in Kavre and Sindhupalchok districts, Nepal: a study on forest resource conditions and the potential for people’s participation 1996 Nepal: Nepal Australia Community Forestry Project

[B81] Craig S Bista G Thomas YA, Karki M, Gurung K, Parajuli D Himalayan healers in transition: Professionalization, identity and conservation among practitioners of *gso br rig pa* in Nepal Proceeding of wise practices and experimental learning in conservation and management of Himalayan medicinal plants 2005 Kathmandu, Nepal: Ministry of Forests and Soil Conservation 411 433

[B82] Kunwar RM Parajuli RR Good governance in natural resource management: A case study from Dolpa district, Nepal Banko Janakari 2007 17 17 24

[B83] Lama YC Ghimire SK Thomas YA Medicinal plants of Dolpo: Amchi’s knowledge and conservation 2001 WWF Nepal, Kathmandu, Nepal: People and Plants Program 150

[B84] Kala CP Current status of medicinal plants used by traditional *Baidhya* in Uttaranchal state of India Journal of Ethnobotany Research & Applications 2005 3 267 27823562803

[B85] Uprety Y Poudel RC Shrestha KK Rajbhandary S Tiwari NN Shrestha UB Asselin H Diversity of use and local knowledge of wild edible plant resources in Nepal J Ethnobiol Ethnomed 2012 8 16 http://www.ethnobiomed.com/content/8/1/16 10.1186/1746-4269-8-16 22546349 PMC3448512

